# What web-based information is available for people with Parkinson’s disease interested in aquatic physiotherapy? A social listening study

**DOI:** 10.1186/s12883-022-02669-3

**Published:** 2022-05-05

**Authors:** Aan Fleur Terrens, Sze-Ee Soh, Prue Morgan

**Affiliations:** 1 Movement Disorder Program, Peninsula Health, VIC, Australia; 2 Department of Physiotherapy, Monash University, VIC, Australia; 3 Department of Epidemiology and Preventative Medicine, Monash University, VIC, Australia

**Keywords:** Aquatic, Physiotherapy, Therapy, Aqua, Hydrotherapy, Parkinson’s

## Abstract

**Background:**

Aquatic physiotherapy is becoming a more frequently utilised treatment for people with Parkinson’s Disease (PD). Consumers are increasingly accessing information regarding health choices online, and it is not known what type or quality of information regarding aquatic physiotherapy is available.

**Methods:**

Web-based platforms (Facebook™, Twitter™, YouTube™, Instagram™, blogs and the web) were searched using the Awario© social listening software. Webpages had to be in English, mention PD, aquatic physiotherapy and its effects. Quality of webpages was assessed using a modified DISCERN tool and content analysis summarised reported effects.

**Results:**

Awario© identified 2992 entries, with 133 assessed using the modified DISCERN tool. A small number (*n* = 31, 24%) described the effects of aquatic physiotherapy for people with PD. Quality of webpages was low, with many lacking information regarding clear sources of information, contraindications to aquatic physiotherapy and descriptions of the therapeutic environment. Content analysis showed several themes; general physical, PD-specific and psychosocial effects. More than a third of webpages indicated that aquatic physiotherapy would improve strength, balance, pain and aid relaxation. A large number (*n* = 96, 72%) described at least one hydrodynamic or hydrostatic property of water, most commonly buoyancy (n-83, 62%).

**Conclusions:**

Overall quality of information was poor, and it is recommended that webpages list all potential contraindications to aquatic physiotherapy and direct consumers to discuss potential participation with their healthcare professionals. Webpages also should include information regarding the therapeutic environment, disclose sources of information and focus on enablers to exercise to improve engagement of people with PD in aquatic physiotherapy.

**Supplementary Information:**

The online version contains supplementary material available at 10.1186/s12883-022-02669-3.

## Introduction

Parkinson’s Disease (PD) is a progressive neurological disorder that can present with both motor and non-motor symptoms [1]. Common symptoms of PD include postural instability, tremor, bradykinesia, rigidity, depression and a stooped posture [2, 3]. It is estimated that over 6.1 million people worldwide have PD, with projections indicating that this figure will rise to 13 million, or more than one percent of the worlds’ population by 2040 [4, 5].

Various exercise modalities are recommended for people with PD to maximise function and independence [6]. Typical physiotherapy treatment includes strength training, gait and balance retraining [7], and interventions such as aquatic physiotherapy have a growing body of evidence to support efficacy in the PD population [8–10]. Aquatic physiotherapy is defined as therapeutic exercise that utilises the hydrostatic and hydrodynamic properties of the water environment [11]. Types of aquatic interventions can vary from aerobic and balance training [9] to programs that focus on core strength [8] and Ai Chi [12]. Several systematic reviews have shown that aquatic physiotherapy is a feasible, safe and effective treatment modality in people with PD [9, 10, 13, 14] and guidelines have recently been published regarding aquatic physiotherapy for the PD cohort [15]. The aquatic environment can however cause orthostatic hypotension or shortness of breath in people with PD, a vulnerable population. Previous literature has shown that swimming is compromised in people with PD [16] and that people with PD have difficulty maintaining a horizontal position in water due to bradykinesia and impaired coordination [17]. It is, therefore important that information regarding the therapeutic environment such as pool depth, water temperature, and staff supervision are fully described and that patients or consumers are aware of any possible contraindications to minimise possible adverse events.

There has been a documented gap between medical information availability and its translation to the consumer, with consumers more recently being keen to have an active role in their own health care [18, 19]. Research has shown that consumers want information regarding medical, physiotherapy and other therapeutic interventions that is clear, concise and easy to understand [20]. In the last 15 years there has been an increase in consumers accessing online information such as web pages to search for information regarding treatment options that are available to inform personal choice, however, there is considerable variability in the type and quality of online information available [21]. Research has also shown that people with PD and their caregivers are increasingly using the internet to gain information to help support their journey with PD [22]. It is thus important as health care providers that we are aware of the type and quality of information that is currently available to consumers online.

A recent study showed that approximately half of consumers looking for health information for themselves reported that decisions regarding their treatment choices were influenced by the results of their online searches [21]. Given the evidence supporting its use, aquatic physiotherapy should be identified as a viable treatment option for those with PD in relevant online webpages searched by consumers. It is also equally important that information available for consumers is accurate to support health literacy. To date, there are no known studies that have identified and evaluated what information is available online for people with PD regarding aquatic physiotherapy.

The primary aim of this study was to evaluate the type and quality of the online information regarding aquatic physiotherapy for PD that is freely available online and targeted at consumers. The secondary aim was to analyse the content of that web-based information.

## Methods

### Search strategy

Data was collected using the social listening tool Awario© from the 8^th^ of January to the 8^th^ of April 2021. Awario© is social listening software that is able to continually search various web-based platforms for key words or phrases in new or previously made posts, with over 13 billion webpages searched daily. Awario© uses the same search term strategy as published literature databases, by combining Boolean operators with AND/ OR to refine results. Awario© searches webpage content for the search terms specified and identifies each entry separately (‘mentions’).

In this study, the following patient, intervention, comparison, outcome (PICO) search strategy was used to search Facebook™, Twitter™, YouTube™, Instagram™, blogs and the web using the following search terms: ((parkinson's OR parkinsons) AND ("aquatic physiotherapy" OR "aqua therapy" OR aquatherapy OR hydrotherapy OR aqua OR "aquatic physio" OR "pool exercise" OR "water exercise" OR "water therapy" OR near/6:aquatic,exercises OR near/6:pool,exercises OR near/6:pool,therapy OR near/6:exercising,water OR near/6:aquatic,therapy OR near/6:aquatic,physiotherapy OR near/6:water,exercises OR near/6:water,therapy) AND NOT ("professional development" OR "police department")) AND lang:en. Addition of the excluded terms professional development (PD) and police department (PD) were added during the search due to the high number of erroneous entries related to these topics. Several websites that had repeated invalid entries were also blocked during the search. All results identified in the search were read to confirm eligibility, with irrelevant and duplicate entries discarded. Two researchers extracted data and verified by sampling a selection of recognised PD consumer websites. Inclusion or exclusion of any contentious mentions were resolved with discussion amongst the research team.

### Selection criteria

All Awario© mentions were read in full to ensure suitability for inclusion. As this study looked to analyse the type and quality of information that exists for people with PD regarding aquatic physiotherapy as a treatment modality, web pages (including Twitter™ posts, Facebook™ posts, YouTube™ videos, Instagram™ stories, blogs and html web pages) were included if they met the following criteria: (1) the term PD was mentioned (2) aquatic physiotherapy (or other synonyms) was mentioned, (3) described the effects (e.g. benefits and contraindications) of aquatic physiotherapy and (4) were written in English. Posts were excluded if they were not aimed towards consumers or were solely timetables for aquatic classes.

### Quality appraisal and data extraction

At the end of the search period, all results from Awario© were exported into a Microsoft Excel spreadsheet to allow for data extraction. Data extracted from Awario© mentions included location by country, type of post (e.g. webpage, blog, tweet etc.), and information on the webpage host (e.g. physiotherapist, health service provider, PD support group etc.). The quality of information was evaluated using a modified version of the DISCERN instrument, which was designed to assess written information regarding treatment choices [23]. A modified version of the DISCERN instrument was used to gather specific information that the original DISCERN instrument did not gather, for example whether webpages described how an intervention works and whether safety and accessibility information was provided. The modified DISCERN had 11 items and could be scored as ‘yes’, ‘no’, ‘partial’ or ‘unsure’ depending on the question. Data were presented in a table, and for each item the most prevalent response was shaded. To assist data interpretation, a traffic light system was used. Thus, if the most prevalent response in webpages was ‘yes’, the box was shaded green, ‘partial’ or ‘unsure’ was shaded amber and ‘no’ was shaded red. A copy of the modified DISCERN tool, and item scoring rules has been provided as Supplementary Information 1. A higher number of ‘yes’ responses in the DISCERN tool indicates a higher quality source of information. Content of webpages was independently assessed by two researchers.

### Evidence synthesis

Webpage content data were summarised using descriptive statistics, and data regarding quality of webpages were displayed visually. Webpage content (i.e. content analysis) pertaining to aquatic physiotherapy was synthesised into categories and subcategories. Any disagreements between researchers regarding DISCERN instrument rating or qualitative coding was resolved by discussion until consensus was reached.

## Results

### Search yield

**Fig. 1:**
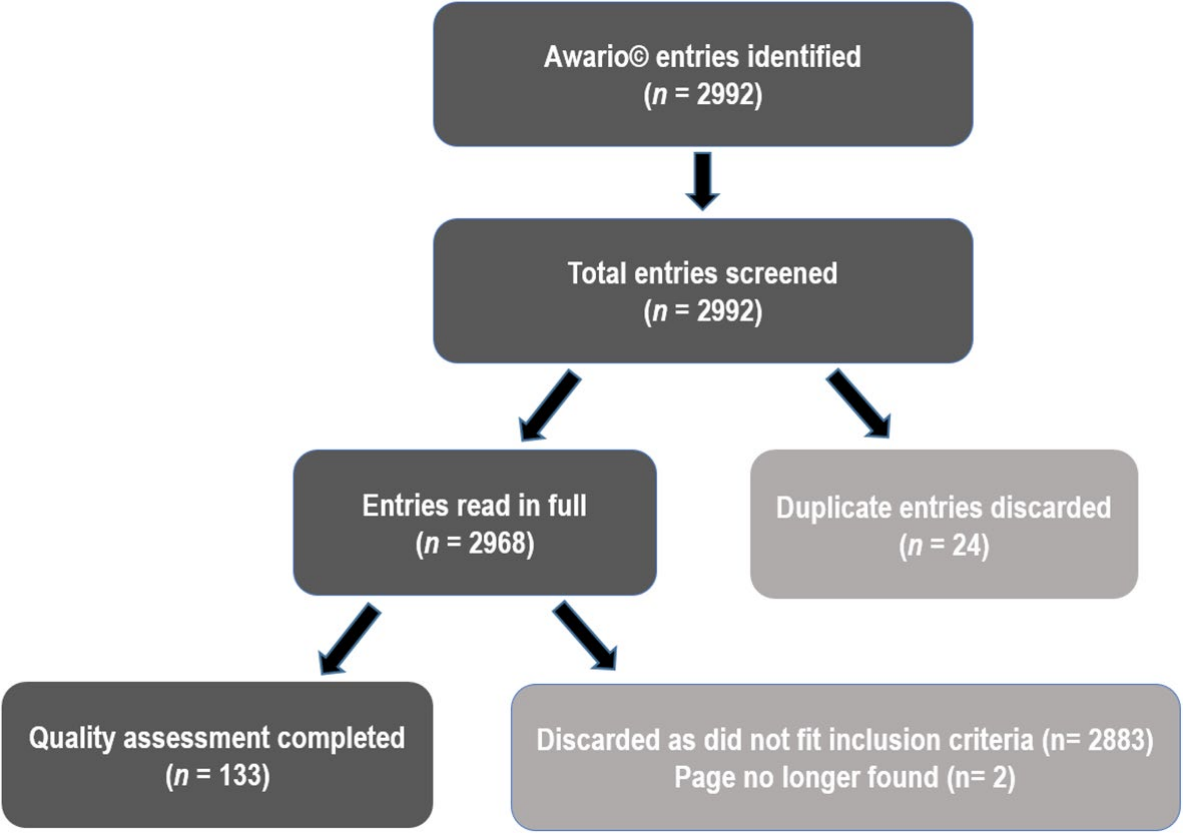
Flow diagram showing web-based entries included in this study

Awario© identified 2992 web-based entries in this search with 2833 entries being discarded immediately as they did not fit the inclusion criteria, and 24 subsequently discarded as they were duplicates (Fig. 1). 133 entries were assessed with the modified DISCERN tool; 83 websites, 2 tweets and 48 news or blog entries. Awario© was able to identify the upload date of 84 entries, and found that the majority of entries were from the past 18 months (*n* = 62, 74%), whilst the oldest entry was from 2013.

**Fig. 2:**
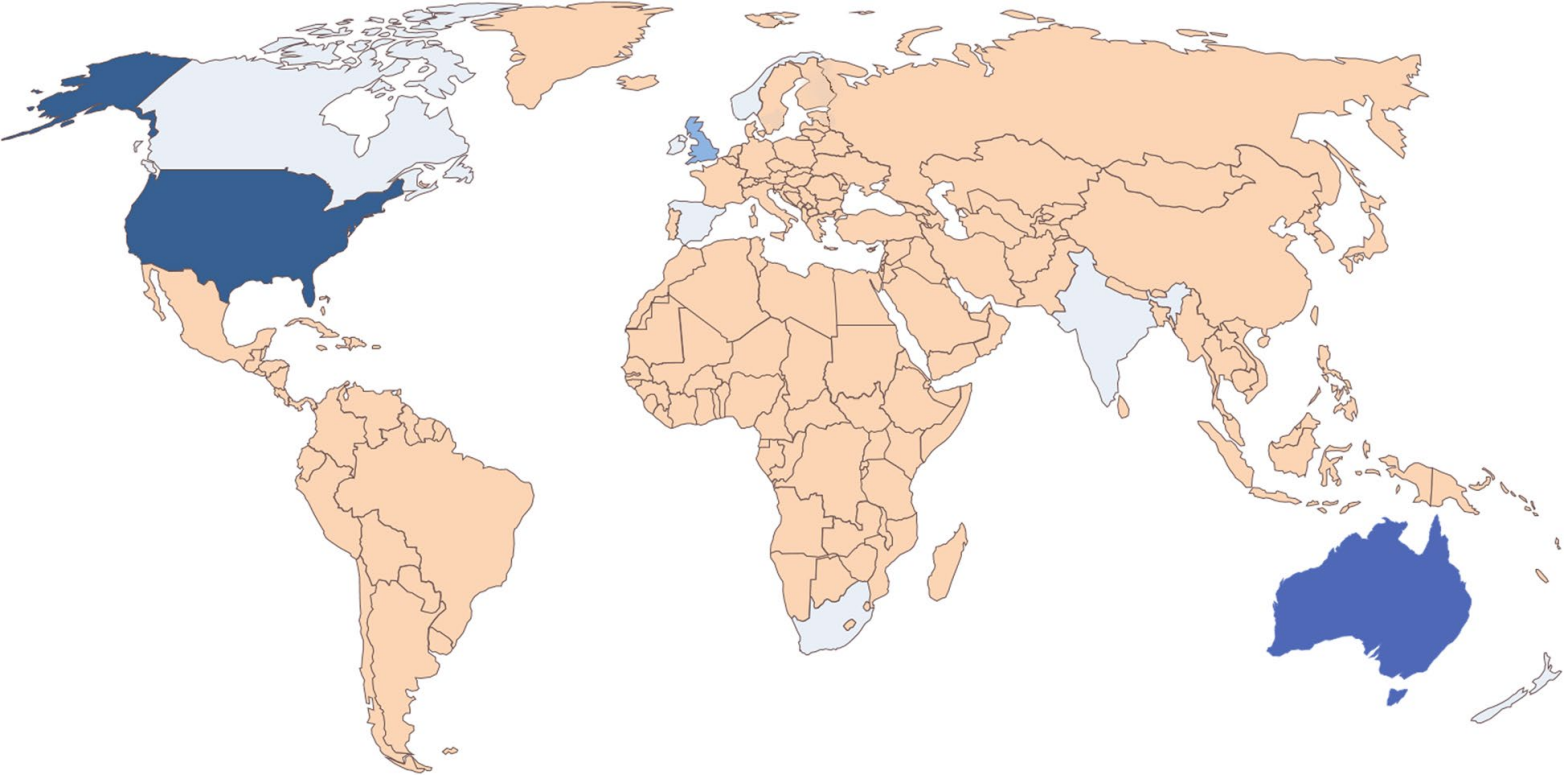
World map showing the distribution of web-based information by country of origin. Darker shades of blue indicate larger numbers of webpages found in the search. Awario found no entries from the countries in beige

Figure 2 shows the country of origin for all entries in the search. The following countries dominated: 61 (46%) from the United States of America, 35 (26%) from Australia, 17 (13%) from Great Britain, seven (5%) from Canada, four (3%) from South Africa (Fig. 2). A further two (1%) entries each were from Europe, India and Ireland, and single entries (1%) from each of New Zealand, Norway and Spain were captured. A large proportion of the webpages were created by private physiotherapists (*n* = 89, 67%) and two webpages (2%) from private biokineticists. There were ten (8%) webpages from professional associations, nine (6%) webpages each from spa or pool companies and public health facilities. There were a further six (5%) private hospital webpages, five (4%) blogs and three (2%) newspaper articles. The terms aquatic physiotherapy and hydrotherapy were most commonly used in the webpage results.

### Quality assessment of webpages

Table 1 displays results of the modified DISCERN instrument. A majority of webpages (*n* = 104, 78%) captured by Awario© were partially relevant to people with PD and were aimed predominantly at the general population, with only a small number (*n* = 23, 18%) of webpages containing information specific to people with PD. The majority of webpages did not disclose the sources of information (e.g. published research or other webpages) to justify the content in their webpage (*n* = 113, 84%) and most webpages identified were deemed to be unbiased (*n* = 119, 89%), with companies promoting pools and spas having the most bias in the information provided. Webpages were deemed as biased if they made statements regarding benefits of aquatic physiotherapy that were not factual or were misleading in order to sell a product.

**Table 1:** Modified DISCERN instrument items

**Item**	**Yes *n* (%)**	**Partial/ Unsure *n* (%)**	**No *n* (%)**
*Is it relevant?*	23 (18%)	**104 (78%)**	6 (4%)
*Are there clear sources of information?*	10 (8%)	10 (8%)	**113 (84%)**
*Is it balanced and unbiased?*	**119 (89%)**	3 (2%)	13 (9%)
*Does it describe how aquatic therapy works?*	**97 (73%)**	-	36 (27%)
*Does it describe the benefits of aquatic therapy for PD?*	31 (24%)	**96 (72%)**	6 (4%)
*Does it describe the contraindications to aquatic therapy?*	3 (2%)	10 (8%)	**120 (90%)**
*Does it describe the therapeutic environment?*	37 (28%)	26 (20%)	**69 (52%)**
*Does it describe the accessibility of the pool?*	26 (20%)	-	**107 (80%)**
*Does it describe the safety aspects of the pool?*	40 (30%)	-	**93 (70%)**
*Is it clear there is more than one possible treatment choice?*	52 (39%)	-	**81 (61%)**
*Does it provide support for shared decision-making?*	16 (12%)	**86 (65%)**	31 (23%)

For each item of the modified DISCERN instrument the most prevalent answer is shown in bold

Only 10% of webpages (*n* = 13) described the potential contraindications to using aquatic physiotherapy in any population, and 24%(*n* = 31) specifically described how aquatic physiotherapy can benefit people with PD. 

The therapeutic environment was poorly described with just 37 (28%) webpages stating exact temperatures or depths of their pools, and 26 (20%) webpages providing a vague reference to either. Water temperatures described ranged from 25° to 40° Celsius (77° to 104° Fahrenheit) and depths ranged from mid torso to 183 cm (6 feet). Accessibility of pools was also poorly reported, with only 26 (20%) webpages describing whether they had adaptive equipment or supports available. Some webpages simply stated that the pool was ‘disability friendly’ whilst others specifically stated that there were ramps, hoists, rails and change rooms close by. Safety, in terms of supervision and assistance available to clients, was also poorly described, with around a third (*n* = 40, 30%) of webpages describing whether aquatic physiotherapy sessions were provided ‘one-on-one’, supervised from the pool deck or fully independent (unsupervised) programs. The m﻿ajority of webpages encouraged consumers to contact the healthcare provider themselves for further treatment advice (*n* = 86, 65%).

### Content of webpages

A large number (*n* = 96, 72%) of webpages described at least one therapeutic property of water and how it would aid in delivering aquatic therapy. The most common therapeutic property reported was buoyancy (*n* = 83, 62%), with temperature or warmth of water being the second most common (*n* = 45, 34%). Similar numbers of webpages stated hydrostatic pressure and resistance (*n* = 23, 18%; *n* = 22, 17%) as properties of water, with few webpages describing water viscosity, turbulence or drag (*n* = 14, 11%; *n* = 12, 9%; *n* = 2, 2% respectively).

Content analysis of the webpage content revealed several themes regarding the effects of aquatic physiotherapy, namely general physical benefits, PD specific physical benefits and psychosocial benefits (Table 2). The benefits of aquatic physiotherapy described by webpages were generally accurate, with a small number (*n* = 5, 4%) sharing unproven information, for example *“aquatic therapy is unparalleled in addressing balance issues”*. General physical benefits were identified in most webpages, with the most common benefits of aquatic physiotherapy correctly proposed as improvement in strength (*n* = 73, 54%), a reduction in pain (*n* = 72, 54%) and improvements in balance (*n* = 65, 49%). Increased range of motion (*n* = 36, 27%), an improvement in swelling or circulation (*n* = 36, 27%) and flexibility (*n* = 32, 24%) were also commonly reported. Other suggested general physical benefits included an improvement in coordination (*n* = 23, 17%), endurance (*n* = 18, 14%), walking (*n* = 15, 11%), fitness (*n* = 12, 9%) and a reduction in spasms (*n* = 13, 9%). A small number of webpages reported potential physical benefits such as better core strength (*n* = 5, 4%), respiratory function (*n* = 4, 3%) and better rotation (*n* = 1, 1%).

**Table 2:** Table illustrating the general physical, PD-specific physical and psychosocial effects of aquatic physiotherapy

Effects of Aquatic Physiotherapy		
*General Physical Effects*	*PD-Specific Physical Effects*	*Psychosocial Effects*
**Better Strength**	Better Posture	**Relaxation/ Reduced Stress**
**Less Pain**	Less Falls	Reduced Fear of Falling
**Better Balance**	Less Dystonia	Improve Mood
Improved Range of Motion	Less Rigidity	Better Quality of Life
Less Swelling/ Improved Circulation	Less Freezing of Gait	More Confidence
Improved Flexibility	Less Constipation	Weight Loss
Improved Coordination	Less Tremors	Reduced Social Isolation
Improved Endurance		Better Sleep
Improved Walking		More Acceptance of Exercise
Less Spasms		
Improved Fitness		
Improved Core Strength		
Improved Respiratory Function		
Improved Rotation		

Webpages could mention more than one effect. Effects mentioned in 30% or more of webpages are in bold. Effects are listed in descending order of mention frequency

Only 39 (30%) of webpages were designed for people with PD and discussed PD-specific benefits when discussing aquatic physiotherapy. These webpages discussed how aquatic physiotherapy may improve balance (*n* = 13, 10%), and therefore reduce the number of falls (*n* = 11, 8%) and episodes of dystonia (*n* = 5, 4%). Other benefits discussed that were specific to people with PD included less rigidity (*n* = 3, 2%), freezing of gait (*n* = 3, 2%), and less constipation (*n* = 2, 1%). One webpage reported that aquatic physiotherapy would improve tremors (*n* = 1, 1%), an area where evidence has not yet been established.

Some webpages also discussed the psychosocial benefits related to aquatic physiotherapy, such as aiding in relaxation and reducing stress (*n* = 50, 37%), a reduced fear of falling (*n* = 14, 10%) and an improvement in mood (*n* = 12, 9%). Some webpages also mentioned that aquatic physiotherapy would result in a better quality of life (*n* = 7, 5%), more confidence (*n* = 7, 5%), weight loss (*n* = 5, 4%), reduced social isolation (*n* = 4, 3%), better sleep (*n* = 1, 1%) and a better acceptance of exercise (*n* = 1, 1%).

## Discussion

This study examined the type, quality and content of webpages regarding aquatic physiotherapy for people with PD. Overall, the quality of webpages were poor, with few webpages created specifically for people with PD and six out of eleven modified DISCERN items unable to be met. A similar study by Morris, et al. [24] found the quality of web-based information regarding boxing as an exercise intervention was also poor, although this study had a small sample of nine web pages.. If, after accessing webpage information, people with PD are not aware that there are potential harmful side effects associated with aquatic physiotherapy or alternatively have not been screened by a medical professional to ensure they are suitable candidates for this treatment modality, they may make an uninformed choice that could lead to harm or injury. Considering people with PD have impaired swimming ability and are unable to maintain a horizontal position in the water or float with ease [16, 17], it is a recommendation from this study that all webpages list potential contraindications to aquatic physiotherapy and also direct people with PD to seek advice from their medical practitioner or physiotherapist to determine whether aquatic physiotherapy is the best therapy choice for that individual.

Although Australia had the second highest number of webpages found it was the only country in the top five results in which the webpage for its national advocacy body for PD did not mention aquatic physiotherapy. It is known that people with PD are advised to access their state or national advocacy body to seek up to date information regarding treatment choices and interventions. Therefore, it is important for national PD bodies to ensure that the information on their webpages reflects current evidence based practice.

We found that there were very few webpages that referred to published research evidence, potentially reflecting the knowledge translation gap identified between research in this area and implementation in practice. There is a large body of evidence supporting aquatic physiotherapy for people with PD and other neurological conditions [9, 10, 11] and the fact that only a small number of webpages referenced research in this area is concerning. Providing evidenced based recommendations for treatment provides a quality check for consumers regarding webpage information and ensures that they are able to make an informed choice regarding what types of treatment they can undertake.

Information regarding the therapeutic environment is poorly reported in published literature [13], and as it was not known whether this information was also reported in webpages, we included these items in the modified DISCERN instrument. Omitting this information may negatively impact on the safe uptake of aquatic physiotherapy for people with PD. Previous research has showed that people with PD prefer to exercise in the aquatic environment under supervision and that more information about local aquatic physiotherapy facilities is needed to improve engagement in this population [25]. It is therefore recommended that consumer webpages include this safety information.

Content analysis of the webpages showed that the most frequently described effects of aquatic physiotherapy for PD were benefits that could be generalised to most medical conditions, such as a reduction in pain and an improvement in strength and balance [26–28]. Few webpages described PD specific evidence based benefits [29, 30] and some made unproven claims, for example, “some diseases can be cured by aquatic physiotherapy”. All print media and web-based information regarding aquatic physiotherapy for people with PD should emphasise enablers such as improvement of balance and reduction in social isolation to improve consumer uptake.

### Limitations

Some limitations from this study need noting. Firstly, the majority of webpage results were from English speaking countries as only English search terms were used. We are aware that aquatic physiotherapy is a popular treatment modality in Italy and Spain and this did not capture these results. Our findings may therefore not apply to people in countries with primary languages other than English. Social media research such as this is relatively new and exploratory, and due to the nature of how Awario© searches webpages, it is not known whether all relevant webpages have been identified. However, hand sampling did confirm the Awario search results. It is also recognised that health services may still produce consumer information regarding aquatic physiotherapy using printed media (i.e. pamphlets or handouts), and that books written on the topic targeting consumers may similarly discuss treatment approaches and effects. Consumers may thus seek information regarding their health needs using print materials that would also not be captured by this study. We also did not incorporate consumers into this work and future studies should do so in order to determine the content and quality of info that should be included in web-based info for people with PD.

## Conclusion

The quality of webpages providing information on aquatic physiotherapy for people with PD was generally poor.. Webpages should include information regarding the therapeutic environment, contraindications and disclose sources of information to improve engagement of people with PD in safe exercise. Content of webpages should focus on enablers to exercise in this population.

## Supplementary Information

**Additional file 1.** 

## Data Availability

The datasets used and/or analysed during the current study is derived from resources in public domains, and further information regarding the data set is available from the corresponding author on reasonable request.
