# Supplementary material for: What web-based information is available for people with Parkinson’s disease interested in aquatic physiotherapy? A social listening study
Source: BMC Neurol. 2022 May 5;22:170. doi: 10.1186/s12883-022-02669-3 (PMC9069763; doi:10.1186/s12883-022-02669-3)
Supplement: Supplementary file 1 — Additional file 1. [file 12883_2022_2669_MOESM1_ESM.docx]

SUPPLEMENTARY INFORMATION

1. **Modified DISCERN instrument**

| Item | Explanation | Scoring |
| --- | --- | --- |
| *Is it relevant?* | **Does the webpage provide information about how aquatic therapy can be used in people with PD?**  Yes= discussed hydrotherapy for PD  Partial = only has PD in a list of conditions that would benefit | Yes/No/Partial |
| *Are there clear sources of information?* | **Does the webpage refer to published research papers?**  Yes = published paper, Partial = websites | Yes/No/Partial |
| *Is it balanced and unbiased?* | **Does the webpage provide an unbiased opinion about the benefits of aquatic therapy i.e. no shock tactics?** | Yes/No/Unsure |
| *Does it describe how aquatic therapy works?* | **Does the webpage provide information about the properties of water?** | Yes/No |
| *Does it describe the benefits of aquatic therapy for PD?* | **Does the webpage provide information about the beneficial effects of aquatic therapy for people with PD?**  Partial = general benefits of aquatic (not PD specific) | Yes/No/Partial |
| *Does it describe the contraindications to aquatic therapy?* | **Does the webpage describe risks / contraindications to aquatic therapy for people with PD?**  Partial = discusses general contraindications/ risks (not PD specific) | Yes/No/Partial |
| *Does it describe the therapeutic environment?* | **Does the webpage describe either the temperature and/or depth of the pool?**  Yes = actual temp and/or depth provided  Partial = vague reference to temp or depth | Yes/No/Partial |
| *Does it describe the accessibility of the pool?* | **Does the webpage describe the accessibility of the pool i.e. ramps, hoists, changing facilities?** | Yes/No |
| *Does it describe the safety aspects of the pool?* | **Does the webpage describe whether the treatment will be supervised by a staff member?** | Yes/No |
| *Is it clear there is more than one possible treatment choice?* | **Does the webpage provide information about other forms of treatment for people with PD?** | Yes/No |
| *Does it provide support for shared decision-making?* | **Does the webpage direct consumers to discuss treatment options with family and/or other health professionals?**  Partial = if directed to contact the site developer | Yes/No/Partial |
